# The development of task sharing policy and guidelines in Kenya

**DOI:** 10.1186/s12960-022-00751-y

**Published:** 2022-07-29

**Authors:** Rosemary Kinuthia, Andre Verani, Jessica Gross, Rose Kiriinya, Kenneth Hepburn, Jackson Kioko, Agnes Langat, Abraham Katana, Agnes Waudo, Martha Rogers

**Affiliations:** 1 Department of Nursing, Emory University, 1520 Clifton Road, Atlanta, GA 30322 USA; 2 U.S. Centers for Disease Control and Prevention, 1600 Clifton Rd, Atlanta, GA 30333 USA; 3 Kenya Ministry of Health, Afya House, Cathedral Road, P.O. Box:30016-00100, Nairobi, Kenya; 4 Emory University Kenya Health Workforce Project, Nairobi, Kenya

**Keywords:** Task sharing, Task shifting, Policy, Guidelines, Human resources for health, Health workforce shortage, Universal healthcare, Kenya, Sub-Saharan Africa

## Abstract

**Background:**

The global critical shortage of health workers prevents expansion of healthcare services and universal health coverage. Like most countries in sub-Saharan Africa, Kenya’s healthcare workforce density of 13.8 health workers per 10,000 population falls below the World Health Organization (WHO) recommendation of at least 44.5 doctors, nurses, and midwives per 10,000 population. In response to the health worker shortage, the WHO recommends task sharing, a strategy that can increase access to quality health services. To improve the utilization of human and financial health resources in Kenya for HIV and other essential health services, the Kenya Ministry of Health (MOH) in collaboration with various institutions developed national task sharing policy and guidelines (TSP). To advance task sharing, this article describes the process of developing, adopting, and implementing the Kenya TSP.

**Case presentation:**

The development and approval of Kenya’s TSP occurred from February 2015 to May 2017. The U.S. Centers for Disease Control and Prevention (CDC) allocated funding to Emory University through the United States President’s Emergency Plan for AIDS Relief (PEPFAR) Advancing Children’s Treatment initiative. After obtaining support from leadership in Kenya’s MOH and health professional institutions, the TSP team conducted a desk review of policies, guidelines, scopes of practice, task analyses, grey literature, and peer-reviewed research. Subsequently, a Policy Advisory Committee was established to guide the process and worked collaboratively to form technical working groups that arrived at consensus and drafted the policy. The collaborative, multidisciplinary process led to the identification of gaps in service delivery resulting from health workforce shortages. This facilitated the development of the Kenya TSP, which provides a general orientation of task sharing in Kenya. The guidelines list priority tasks for sharing by various cadres as informed by evidence, such as HIV testing and counseling tasks. The TSP documents were disseminated to all county healthcare facilities in Kenya, yet implementation was stopped by order of the judiciary in 2019 after a legal challenge from an association of medical laboratorians.

**Conclusions:**

Task sharing may increase access to healthcare services in resource-limited settings. To advance task sharing, TSP and clinical practice could be harmonized, and necessary adjustments made to other policies that regulate practice (e.g., scopes of practice). Revisions to pre-service training curricula could be conducted to ensure health professionals have the requisite competencies to perform shared tasks. Monitoring and evaluation can help ensure that task sharing is implemented appropriately to ensure quality outcomes.

## Background

Human resources for health (HRH) is an essential health system building block, and the World Health Organization (WHO) advises that health systems have adequate HRH engaged in service delivery to improve population health [1]. To attain the Sustainable Development Goals (SDGs), the WHO recommends a health workforce density of 44.5 doctors, nurses, and midwives per 10,000 population [2]. However, there is a chronic shortage of health workers globally; the *Global Strategy on Human Resources for Health: Workforce 2030* reports an estimated global needs-based shortage of over 17 million health professionals, including over 9 million nurses and midwives and 2.6 million physicians. The greatest shortages are in regions with the highest unmet health needs, such as South East Asia and sub-Saharan Africa [2].

The chronic HRH shortage makes it difficult to provide universal HIV services, attain the SDGs, and enhance population health. The global burden of disease is increasing as populations are living longer [3]. Additionally, low- and middle-income countries (LMICs) are facing the double-burden of infectious diseases and rising prevalence of non-communicable diseases, leading to an increased demand for access and provision of health services [4].

In Kenya, the number of active healthcare workers remains far below the current WHO recommendations. As reported in the *Kenya Health Workforce Report*, Kenya has 53,118 active doctors, clinical officers, and nurses and midwives; thus, the health worker to population ratio in the country is 13.8 providers per 10,000 individuals in the nation’s population [5]. This ratio is less than one third the WHO critical threshold recommendation of 44.5 providers per 10,000 individuals. As in many LMICs, Kenya’s health workforce largely comprised nursing professionals; Kenya has 8.3 nurses per 10,000 population compared to WHO’s recommendation of 25 nurses per 10,000 population [5].

Kenya has made strategic investments to scale-up the nursing workforce, including increasing the national capacity to train nursing professionals by focusing on the expansion of nurse training institutions. This approach led to a 32.5% increase in the number of nursing schools, from 77 to 102 between 2006 and 2015 [5]. This expansion subsequently led to increased student enrollment into nursing programs, either as new entrants or advanced practice [5]. Additionally, the United States President’s Emergency Plan for AIDS Relief (PEPFAR) has supported training for thousands of health workers [6]. Despite these investments, Kenya’s healthcare workforce shortage has persisted.

Kenya has a large number of people living with HIV (PLHIV), which has strained the healthcare system and its workforce. In 2016, when the 2017–2030 Kenya Task Sharing Policy and Guidelines (TSP) [7]. were being developed, there were 1.6 million PLHIV, 62,000 new infections, and 36,000 AIDS-related deaths in Kenya [8]. Given the scarcity of physicians, the model of physician run clinics, common in high income countries, is not feasible for Kenya and other LMICs [9].

In light of the global disease burden, HIV pandemic, and critical shortage of trained health workers, in 2007 the WHO released guidelines on *task shifting* as one approach to address HRH concerns and increase access to HIV care and other health services. According to WHO, task shifting is “a process whereby specific tasks are moved, where appropriate, to health workers with shorter training and fewer qualifications” [10]. If implemented appropriately, task shifting is intended to improve health care coverage by utilizing more widely available cadres, such as nurses, clinical officers, and community health workers, to improve the efficiency of already existing HRH. After the release of WHO’s task shifting guidelines, the term *task sharing* was formally introduced in the scientific literature by the Institute of Medicine (IOM) in 2010. The IOM introduced the concept of task sharing as a strategy for capacity building, prevention, treatment, and care of HIV/AIDS in Africa. Task sharing addresses bottlenecks in the delivery of health services through efficient use of existing HRH, whereby “physicians, nurses, dentists, and other health professionals delegate health care responsibilities and relevant knowledge to others, including community health workers” [11]. In addition to encouraging collaboration, the IOM recommended that task sharing focus on the promotion of competency-based training for health workers taking on new tasks.

*Task shifting* and *task sharing* are used in a variety of public health settings to meet the demand for health services and address workforce shortages. Although WHO initially recommended task shifting in the context of addressing the HIV epidemic, its application was extended to address other areas such as maternal and newborn health care [12] with WHO’s maternal and newborn task shifting guidelines [13]. Task shifting has been implemented to support reproductive health services [14] and tuberculosis care [15]. The use of task sharing as a model of delivering care is gaining popularity in under-resourced regions [15]. However, implementation was often informal and established organically to adapt to HRH shortages [16]. Task sharing is used widely in sub-Saharan Africa, increasing access to healthcare services, and yielding positive health outcomes [17, 18].

However, despite the benefits of task sharing, and its widespread use in across Africa, there is a scarcity of evidence documenting the process of developing and implementing task sharing policies and guidelines. Guidelines and policies, informed by stakeholders, may facilitate the process of task distribution and alleviate the workforce burden among health workers in an organized and systematic manner.

Policy development has been described in the public policy literature as one of multiple stages in the policy cycle [19]. Policy adoption, policy implementation, and policy evaluation are examples of other stages in the policy cycle which sequentially follow policy development. The process of policy advancement is relevant to HIV services and health generally [20], as exemplified by the case of task sharing policy development in Kenya.

Stakeholders have been defined as, “the individuals, organizations, and even governments involved in policy-making, the processes related to policy development and implementation, and the interactions between them” [21]. A key principle in health policy is that of stakeholder engagement, as summarized here: “Successful implementation of healthcare interventions relies on stakeholder engagement at every stage” [22]. However, the practice of stakeholder participation is complex and challenging [23].

## Case presentation

This section describes the multi-year process whereby the Government of Kenya, with stakeholder participation, drafted then adopted Kenya’s Task Sharing Policy and Guidelines. Several of us were active participants in this process; we hope that by sharing insights into the TSP development process, others will be better informed to advance task sharing.

As public health practitioners, our purpose was to support drafting and adoption of task sharing by the Government of Kenya, in order to advance pediatric HIV treatment, other HIV services, and additional health services as appropriate. It is important to note that publication in a peer-reviewed journal was a secondary consideration, which we only embarked upon after the TSP was adopted and disseminated for implementation. This has implications for the approach described in this article. For example, our desk review on task sharing was conducted rapidly in time for an in-person meeting of stakeholders as opposed to being conducted over several months with greater detail and methodological rigor as might be more common for the purpose of publication. In short, this article describes the TSP development and implementation process as it played out on the ground in Kenya from 2015 to 2019.

In 2015, Emory University, in collaboration with the Kenya Ministry of Health (MOH), the U.S. Centers for Disease Control and Prevention (CDC), and PEPFAR sought to advance task sharing to promote equitable access to universal health coverage (UHC), including HIV services at the national, county, sub-county and community levels in Kenya. Early in this process, the MOH partnered with several institutions to establish the Project Advisory Committee (PAC) overseeing the development of the TSP. As stated in the TSP, it is intended to “facilitate enhanced quality service delivery in Kenya through the implementation of an integrated task sharing framework, improving access to essential health services, including HIV/AIDS prevention, care and treatment” [14, 24].

The process of development, adoption, and implementation of the Kenya TSP occurred in five phases which are described in detail below.

### Phase 1 (February 2015–September 2015)—funding and partnering

CDC allocated funding through the PEPFAR Advancing Children’s Treatment initiative to facilitate development of Kenya’s TSP, since the lack of advanced practitioners trained and authorized to provide HIV treatment to children had been identified as a barrier to PEPFAR’s HIV treatment coverage for children in Kenya. In February 2015, CDC Kenya and Kenya’s nursing professional leaders began to conceptualize the formalization of task sharing (as recommended by WHO) to address HRH challenges in the country. In August 2015, the Emory University Kenya Health Workforce Project met with the Kenyan Director of Medical Services (DMS) from the MOH who provided high-level support to develop the TSP guidelines and policy. The Kenya MOH then identified key members for the establishment of a Policy Advisory Committee (PAC) to steer the initiative. PAC stakeholders included but were not limited to the Kenya MOH, Kenya’s National AIDS and STI Control Programme (NASCOP), county governments (which implement health services under Kenya’s Constitutionally mandated devolution), training institutions, health professional regulatory bodies for nurses, physicians, clinical officers, laboratorians, and other cadres, non-governmental organizations (NGOs) and faith-based organizations (FBOs), and partner agencies such as the WHO, United States Agency for International Development (USAID), and CDC (see Appendix A for a comprehensive list of PAC members).

### Phase 2 (September 2015–October 2015)—stakeholders set out process and policy scope

In September 2015, the PAC held its first meeting to identify the best approach for the development of the task sharing guidelines and policy. The PAC worked collaboratively to ensure PAC representation of key stakeholders, as well as to identify the desired scope of the task sharing policy, the best approach for policy development, and the level of approval needed in the Kenya MOH.

In preparation for the first meeting of the PAC, Emory and CDC collaboratively conducted a rapid desk review which included evidence-based guidance from WHO, recent task sharing policies in other neighboring countries (including scopes of practice for various healthcare cadres), and peer-reviewed research, as well as a mapping exercise of health workers and unmet ART needs. Materials reviewed were in English and Portuguese.

The mapping exercise utilized data from Kenya’s Human Resources Information System (HRIS), an interoperable database connecting several health professional regulatory bodies with their members to facilitate registration, licensing, continuing professional development (CPD), and other regulatory functions [27]. Overall, the proportion of nurses per population was found to be three times that of clinical officers which in turn was double that of medical officers. With six times more nurses than physicians, the importance of nurses to HIV treatment and the country’s inability to rely on physicians alone or in conjunction with clinical officers who are also far outnumbered by nurses, became clear to the stakeholders present. Complementary data sources were the 2010 Kenya Service Provision Assessment [25], which showed that fewer than 40% of nurses in Kenya were prescribing ART.

The rapid desk review summarized key points from WHO task shifting guidelines [10], national guidance and policies (e.g., guidelines for antiretroviral therapy in Kenya [26]), and peer-reviewed publications on task sharing/shifting. WHO task shifting recommendations that were highlighted in the rapid desk review are summarized in Table 1.

**Table 1:** WHO task shifting recommendations included in the desk review

WHO recommendation number	Area of focus	Recommendation
#1	Strengthening task sharing	Implementing a task shifting approach is recommended in countries where access to healthcare services is limited due to health workforce shortages
#3	National harmonization	A nationally endorsed framework is recommended for countries opting to implement task sharing
#16	Types of task sharing	Countries that implement a task sharing approach should adopt task sharing models that are best suited to the context
#17	Efficient referral systems	There should be efficient referral systems in place to facilitate implementation of task sharing and the health workforce should be trained to use the referral systems appropriately
#18	Safe and effective delivery of clinical tasks by non-physician clinicians	Some clinical tasks can be conducted effectively by trained non-physician clinicians
#19	Safe and effective delivery of clinical tasks by nurses and midwives	Nurses and midwives can undertake HIV clinical services including ART initiation and management
#20	Safe and effective delivery of HIV counseling, education, and other services by community health workers (CHWs)	CHWs and PLWHIV can provide HIV services in health facilities and community settings
#21	Self-care and support for others by people living with HIV	PLWHIV can be empowered to take responsibility for their care and also support their peers
#22	Task shifting of diagnostic and dispensing services	Pharmacy, laboratory professionals, and non-clinical staff such as record managers and administrators can provide health services and should be included in task sharing

To further ground the rapid desk review in the national and regional policy reality, recent scopes of practice or schemes of service were reviewed from nearby countries including the 2014 Tanzania Nursing and Midwifery Council Scope of Practice for Nurses and Midwives [27], the 2014 Uganda Scope of Practice for Nurses and Midwives [28], the 2012 Scope of Practice for Nurses in Kenya [29], and 2011 Guidelines for ART in Kenya [26], which describes the roles and responsibilities of clinical officers. Highlights included: (i) Tanzania’s general authorization of registered and enrolled nurses to prescribe and administer medicines according to their specific scopes of practice, (ii) Uganda’s indication that nurses and midwives should not perform tasks outside their respective scopes of practice, (iii) Kenya’s clinical officer scope of practice authorizing all 10 grades of public sector clinical officers to initiate and manage ART, and (iv) Kenya’s nursing scope of practice generally authorizing prescription of drugs for acute and chronic illnesses. Concurrently, the 2011 Kenya ART guidelines acknowledged that actual staffing varies from staffing norms and that task shifting allows for best use of available health staff, all of whom it called for to be trained in HIV care and treatment. Being more specific with regard to who could provide ART, Malawi’s 2011 integrated HIV service guidelines [30] stated that, “all certified clinical PMTCT/ART providers are authorized to prescribe and dispense ART (Doctors, Clinical officers, Medical Assistants, Registered Nurses, Nurse/Midwife Technicians)”. Other policies supportive of task sharing were reviewed from the Democratic Republic of the Congo, Eswatini, and Mozambique.

Published, peer-reviewed data presented to the PAC in the rapid desk review included: a systematic review finding equal or improved HIV clinical outcomes with nurse managed care [31], positive stakeholder perspectives on task sharing such as reduced waiting times and improved access [32], reduced patient out-of-pocket costs with a task shifting home-based care model [33], a survey finding 11 of 15 countries in east and southern Africa practicing nurse-initiated and managed ART with Kenya being one of the four in the minority [34], and over 70% of community members surveyed in Malawi and Uganda supporting task shifting from doctors to nurses and from nurses to community health workers [35]. In sum, the review identified gaps in the existing models used to deliver healthcare in Kenya and identified opportunities to utilize the competency and skills of various cadres more effectively through task sharing.

At its first meeting, the PAC decided to develop a task sharing policy inclusive of HIV care and other health services essential for UHC. This initial decision to develop a task sharing policy that included but was not limited to HIV services led to a lengthier, more complex process involving stakeholders across diseases and conditions in order to produce policy and guidelines documents highly relevant to Kenya’s overall goal to achieve UHC, as well as PEPFAR’s more focused goal to expand HIV services.

The PAC subsequently established five technical working groups (TWGs) and suggested TWG members to advance the development of the Kenyan TSP within their respective thematic areas. Table 2 provides a summary of the five TWGs and their roles in the development of the TSP. TWG members included representatives from the national MOH, county level health offices, health professional regulatory bodies including those for physicians, clinical officers, nurses, pharmacists, and laboratorians, as well as health training institutions, FBOs, NGOs, Emory, USAID, CDC, and WHO. To emphasize the importance of the TSP, the Kenya Principal Secretary for Health officially launched the TWGs in September 2015. The PAC recommended areas of concentration for the TWGs informed by the desk review.

**Table 2:** Summary of the technical working groups and assigned roles

Technical working group (TWG)	Role
TWG #1: Introduction and Evidence	Conducted a mapping exercise and focused on reviewing evidence and background information regarding task sharing policy in Kenya’s health system. This group worked together to provide the aim and objectives of the TSP and to conduct a situational analysis
TWG #2: Legal and Regulatory	Researched and analyzed existing laws, regulations, and policies pertinent to task sharing and recommended harmonization of national laws, policies, regulations, and guidelines in support of task sharing
TWG #3: Training	Focused on identifying requirements to equip health workers with the necessary knowledge, skills, and competencies to provide essential healthcare services. Stakeholders conducted a task analysis of each cadre, identifying their training needs, then collaborating to explore best practices to promote quality healthcare through pre-service, internship, in-service training, CPD
TWG #4: Service Delivery	Worked together to identify key service areas and ensure the service delivery guidelines were comprehensive for all six levels of Kenya’s healthcare system
TWG #5: Implementation, Monitoring and Evaluation	Applied evidence-based principles of monitoring and evaluation to develop an implementation checklist, identify data collection methodologies, and develop indicators to monitor the progress of TSP implementation

### Phase 3 (October 2015–May 2016)—technical working groups draft the TSP

The TWGs convened periodically over the course of a year to develop the TSP. The groups utilized an iterative process involving three collaborative rounds of in-person meetings and online communication to develop the policy. Round 1 of the TWG meetings took place from October 27th to November 4th, 2015*.* During this time, the groups developed a draft outline for the policy. Round 2 of the TWG meetings, which took place from December 1st to 9th, 2015, resulted in the development of an initial draft of the TSP policy, with each TWG drafting its respective section. Revision of the initial draft took place from February 2nd to 9th, 2016 during round 3 of the TWG meetings. For about three months after the round 3 meetings, the team collaborated to complete several activities that led to finalizing the policy. These activities included: finalizing the comprehensive TSP guidelines, reviewing of the guidelines with healthcare workers (HCW) who were members of the PAC, revision of policy using feedback from HCWs, and convening of the PAC to finalize the policy. PAC members reviewed the TSP to ensure integration and coherence, since the policy was drafted section-by-section by the respective TWGs. The final TSP policy and guidelines were submitted to the Kenya MOH on May 15th, 2016.

### Phase 4 (May 2016–May 2017)—finalization and adoption by Government of Kenya

After the PAC’s submission of the draft documents to the MOH and finalization by MOH staff, the MOH Cabinet Secretary and DMS reviewed, and approved the TSP. MOH finalization included division of the draft TSP into two separate documents: (i) an overarching policy document, and (ii) a more detailed and operationally focused guidelines document. To further emphasize the importance of the task sharing policy and guidelines, an official launch took place during a ceremony involving the MOH’s highest leadership, CDC Kenya officials, county health officials, health professional regulatory board representatives, and other stakeholders. Thereafter, the policy and guidelines were disseminated to the counties.

### Phase 5 (May 2017–April 2019)—implementation of policy and guidelines

The fifth phase of the process is implementation, monitoring, and evaluation. Activities to facilitate this final phase included Emory University in collaboration with the MOH disseminating the TSP and guidelines. TSP sensitization to increase awareness of the policy and guidelines initially took place in 10 select counties, with plans to sensitize health representatives from all 47 counties in Kenya. However, in June of 2017, only one month after the TSP was launched by the Government of Kenya, the Association of Kenya Medical Laboratory Scientific Officers (AKMLSO) asked Kenya’s High Court to stop its implementation. It is not the purpose of this paper to delve into the legal arguments made either by the AKMLSO or the Kenya MOH. Although it is important to note that the High Court decided to stop TSP implementation in its decision of April 2019 [7].

### Task sharing policy and guidelines

The TSP development process resulted in the development of the Kenya 2017–2030 task sharing policy and guidelines [7] that were briefly implemented then stopped by the judiciary. The policy document provides a brief general orientation to task sharing and key aims of task sharing policy. The guidelines list priority health-related task sharing activities by cadre with references to the evidence base. The guidelines are organized into six chapters which are summarized in Table 3.

**Table 3:** Summary of the Kenya 2017–2030 task sharing policy and guidelines

Chapter	Description of chapter
Chapter 1: Introduction	Presents background information on Kenya’s health indicators, overview of the health system, and the health worker shortage crisis; thus, making a case for why introducing task sharing in Kenya would be beneficial to addressing the workforce shortages. The chapter also discusses the global evidence-based recommendations for task sharing from the WHO
Chapter 2: Legal, Regulatory and Policy Framework	Highlights various Kenyan laws, regulations and policies, and reviews whether they enable or restrict the implementation of task sharing in the country, while urging harmonization of Kenya’s laws, regulations, and other policies in support of task sharing
Chapter 3: Training and Education	Presents an overview of health training in Kenya and summarizes the training provided to select cadres and addresses the need for specialized training and continuing education for these cadres to facilitate implementation of task sharing
Chapter 4: Task Sharing by Cadre and Level	Presents targeted cadres and a large number and variety of tasks that may be shared by each cadre after ensuring competency to perform the task. See Appendix B for a list of cadres targeted for task sharing To facilitate implementation, the information is presented in tables that are easy to read and interpret. These tables constitute approximately half of the entire document, as several hundred tasks are listed alongside the evidence base for sharing each task with each cadre. These tasks include HIV rapid testing, HIV treatment, HIV Pre-Exposure Prophylaxis, medical male circumcision, TB case identification, malaria rapid diagnostic testing, micronutrient supplementation, and provision of immunizations, among many others. See Appendix C for an example of the HIV testing and counseling table included in the TSP document
Chapter 5: Monitoring and Evaluation (M&E)	Describes the areas that M&E of the TSP will cover as well as the guiding principles and M&E framework of the TSP
Chapter 6: Recommendations	Makes suggestions for the way forward, centering around five key areas: (i) adoption and implementation, (ii) harmonization of laws, policies and regulations, (iii) training, (iv) service delivery, and (v) monitoring and evaluation of TSP implementation

## Discussion

The process of developing the TSP guidelines and policy encouraged collaboration and consensus among the stakeholders. Consensus was promoted by convening broadly representative groups of stakeholders with varying roles in Kenya’s health care system (PAC and TWGs), through high-level MOH leadership delegating responsibility to the PAC and TWG members for policy development, and by frequent in-person meetings of the TWGs over the course of a year. Email was used for communication in between in-person meetings; however, in-person meetings were more effective than virtual communications at advancing consensus and policy development.

As noted earlier, stakeholder participation is an important principle than can be difficult to put into practice. As seen with Kenya’s TSP, each context in which policy development and stakeholder consensus takes place exerts a unique influence on the outcome. In Kenya, various stakeholders participated in agreeing on the need for a policy, scoping the policy, and developing the policy. However, one stakeholder group challenged the policy after it had been adopted and effectively stopped its implementation through the courts, at least temporarily. In hindsight, a systematic stakeholder analysis at the beginning of the process might have mitigated such a risk.

As noted in the TSP, there are several pending items to be addressed to advance task sharing. Revision of certain legislation in Kenya may be considered to lift restrictive laws prohibiting the sharing of certain tasks. For example, as noted in the guidelines document, the Public Health Act restricts disease reporting to medical officers even though many health facilities in Kenya lack even one medical officer. Further, The Clinical Officers Act does not authorize HIV treatment by private sector clinical officers.

Revision of scopes of practice and schemes of service for the various cadres delivering healthcare services may also be considered. Tasks that can be performed competently may nevertheless not be covered by the cadre’s scope of practice. Regulatory authorities could, therefore, work collaboratively to address how all cadres can practice to the full scope of their training. In some cases, cadres in under-resourced areas are practicing beyond their official scope of practice because of clients’ needs; however, there may be the risk of liability for performing certain tasks, even if performed competently.

Capacity development of stakeholders is key for successful task sharing implementation [36]. Ideally, capacity building would take place across public and private sector workplaces, professional association forums, and training institutions. For already credentialed health workers, training institutions could consider incorporating CPD and continuing medical education (CME) to increase knowledge, skills, and competencies to facilitate task sharing. To meet the needs of the future healthcare workforce, the TSP encourages training institutions to revise the existing training curricula at all levels to include new tasks supportive of task sharing.

Many challenges arose during the process of TSP development, adoption, and implementation. The TSP policy and guidelines development demanded time, resources, and dedication of key individuals from the MOH. The MOH is faced by many competing priorities, some of which had potential to be impacted by TSP process. However, adequate preparation by parties interested in furthering task sharing prior to the meeting with the MOH facilitated buy-in. In addition, TSP project champions with prior experience in the MOH secured key meetings to gain support and advance policy development with MOH leadership.

As with other collaborative processes, engaging all stakeholders and coordinating the logistics to identify meeting times suitable to all parties is challenging. Oftentimes, senior officials involved in the TWGs had competing tasks and could not participate in meetings. Some leaders designated their assistants as delegates in their absence. Individuals unable to join received meeting briefings. Although health professional member associations were not directly involved in the development of the policy, the councils responsible for regulating varied health professionals (including physicians, clinical officers, pharmacists, nurses, and medical laboratorians) were directly involved in TSP development.

The process of developing the task sharing policy and guidelines was complex and rigorous, requiring dedication and commitment from all stakeholders. A major barrier during the policy development process was the nationwide nurses and doctors strikes that lasted for months. The strikes took attention away from the TSP and some of the TWG meetings had to be delayed because MOH senior management representatives were dealing with the strike issues.

An ongoing challenge is the Kenyan court ruling in April of 2019 in the case brought by the AKMLSO against the MOH wherein the Kenya High Court stopped implementation of the TSP [37]. Despite evidence-based WHO guidance supporting task sharing of diagnostic services with other trained health workers including clinical officers, nurses, and community health workers (as detailed above), the legal challenge from a laboratorian professional association has stopped the TSP.

During the TSP development process, many lessons were learned that may provide insights to inform task sharing policy development, adoption, and implementation in other countries. High-level political will from the MOH and involvement of a wide range of stakeholders from the health sector promoted ownership and buy-in. During the collaborative process, participating stakeholders identified that sharing roles among the TWGs ensured a focused and faster process. To advance collaboration, the TSP team shared progress reports with the human resources for health interagency coordinating committee in Kenya, on which the counties are represented, helping to further secure essential county level input.

The TSP PAC engaged the MOH review team early in the process and received orientation on the flow of government policy, which enhanced the efficiency of the policy development process and enabled the PAC to meet the time constraints discussed above. While technology facilitates remote meetings in many parts of the world, the TWGs found that meeting in person was most productive, and much progress was made during the in-person meetings.

## Conclusions

The development of a national task sharing policy established a framework in Kenya for innovative and differentiated service delivery models for essential health services. The TSP (if implementation is allowed to resume by the Kenyan courts) could help to advance task sharing in Kenya and address the workforce shortages. Crucial to renewed implementation would be to follow-up on revisions of legislation, scope of practice, capacity building, schemes of service and curricula.

## Figures and Tables

**Fig. 1:**
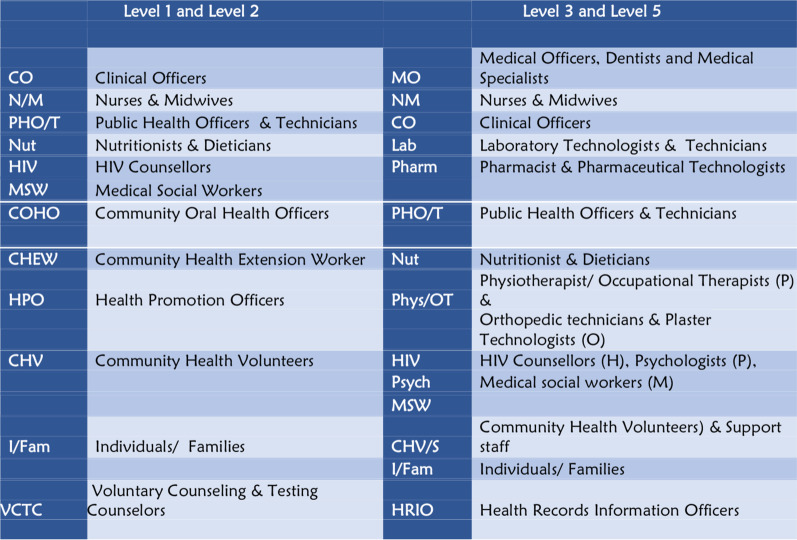
Cadre abbreviations. Reprinted from *Task Sharing Policy Guidelines 2017–2030* (p. 53) by Kenya Ministry of Health, Nairobi, Kenya. Copyright 2017 by the Principal Secretary, Ministry of Health, Kenya. Reprinted with permission

**Fig. 2:**
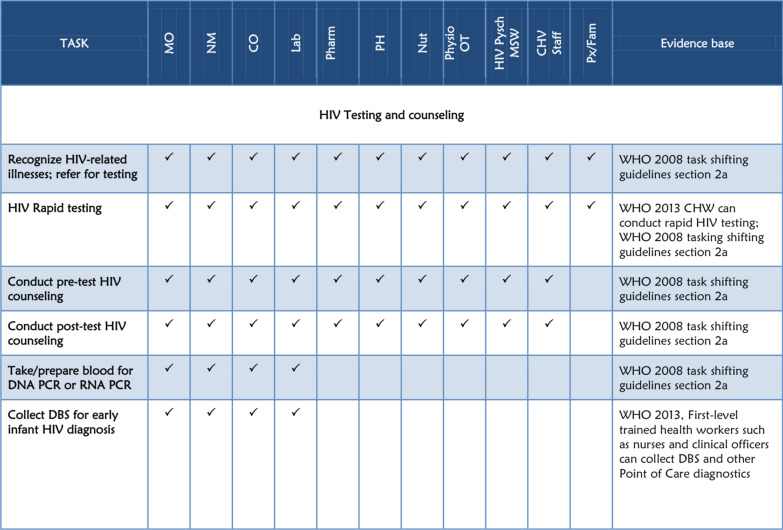
HIV testing and counseling tasks that may be conducted with adequate training and supervision. Reprinted from *Task Sharing Policy Guidelines 2017–2030* (p. 85) by Kenya Ministry of Health, Nairobi, Kenya. Copyright 2017 by the Principal Secretary, Ministry of Health, Kenya. Reprinted with permission

## Data Availability

The data that support the development of the Kenya TSP documents are available from the Kenya Ministry of Health. Summaries of the findings were published in the TSP documents, which are publicly available and can be accessed via the following hyperlink: https://www.hesma.or.ke/wp-content/uploads/2017/02/Task-Sharing-Guideline-2017.pdf

## Appendix A

### Comprehensive list of PAC members

**Table:**

Member	Institute of Affiliation
Dr. Nicholas Muraguri	Kenyan Ministry of Health (MOH)
Dr. Izaq Odongo	Kenyan Ministry of Health (MOH)
Dr. Hannah Wamae	Kenyan Ministry of Health (MOH)
Mr. Joseph Mirereh	Kenyan Ministry of Health (MOH)
Dr. Santau Migiro	Kenyan Ministry of Health (MOH)
Dr. Pacifica Onyancha	Kenyan Ministry of Health (MOH)
Dr. Rachel Nyamai	Kenyan Ministry of Health (MOH)
Dr. Martin Sirengo	National AIDS and STIs Control Program (NASCOP)
Dr. Irene Mukui	National AIDS and STIs Control Program (NASCOP)
Dr. Peter Kimuu	Kenyan Ministry of Health (MOH)
Dr. Jackson Kioko	Kenyan Ministry of Health (MOH)
Professor Issaac Kibwage	University of Nairobi (UoN)
Mr. David Njoroge	Kenyan Ministry of Health (MOH)
Dr. Andrew Mulwa	County Executives Committee (CEC) for Health
Dr Jack Magara	County Directors of Health Services (CDHS)
Mrs. Agnes Waudo	Emory Kenya Health Workforce Project
Mr. Sylvester Kimaiyo	Academic Model Providing Access to Healthcare (AMPATH)
Mr. Meshack Ndolo	IntraHealth International
Dr. Janet Muriuki	IntraHealth International
Mr. Mathew Thuku	IntraHealth International
Mr, Mark Hawken	International Center for AIDS Care and Treatment Program (ICAP)
Mrs. Susan Otieno	Kenyan Ministry of Health (MOH)
Mr. Andre Verani	Centers for Disease Control and Prevention (CDC) Atlanta
Mr. James Kwach	Centers for Disease Control and Prevention (CDC) Kenya
Dr. Abraham Katana	Centers for Disease Control and Prevention (CDC) Kenya
Dr. Elly Odongo	Centers for Disease Control and Prevention (CDC) Kenya
Mr. Peter Waithaka	United States Agency for International Development (USAID) Kenya
Mrs. Edna Tallam Kimaiyo	Nursing Council of Kenya (NCK)
Mr. Micah Kisoo	Clinical Officers Council (COC)
Mr. Daniel Yumbya	Kenya Medical Practitioners and Dentists Board (KMPDB)
Dr. Samuel Mwenda	Christian Health Association of Kenya (CHAK)
Ms. Jacinta Mutegi	Kenya Conference of Catholic Bishops (KCCB)
Ms. Firdaus Omar	Supreme Council of Kenya Muslims (SUPKEM)
Mr. Peter Tum	Kenya Medical Training College (KMTC)
Dr. Jane Karonjo	Mt. Kenya University
Professor Barasa Otsyula	Kenya Methodist University (KeMU)
Mrs. Jessica Gross	Emory University/ Centers for Disease Control and Prevention (CDC) Atlanta
Custodia Mandlhate	World Health Organization (WHO) Kenya
Dr. Nduku Kilonzo	National AIDS Control Council (NACC)
Dr. Celestine Mugambi	National AIDS Control Council (NACC)
Prof. Sylvia Ojoo	University of Nairobi
Dr. Agnes Langat	Centers for Disease Control and Prevention (CDC) Kenya

## Appendix B

See Fig. 1.

## Appendix C

See Fig. 2.

## Abbreviations

HRH: Human resources for health
WHO: World Health Organization
SDG: Sustainable Development Goals
LMICs: Low- and middle-income countries
PLHIV: People living with HIV
IOM: Institute of Medicine
MOH: Ministry of Health
CDC: Centers for Disease Control and Prevention
UHC: Universal health coverage
TSP: Task sharing policy and guidelines
PEPFAR: President’s emergency plan for AIDS relief
DMS: Director of medical services
PAC: Policy advisory committee
USAID: United States Agency for International Development
TWG: Technical working groups
NASCOP: National AIDS and STI Control Programme
CPD: Comprehensive continuing professional development
HCW: Healthcare workers
CME: Continuing medical education
